# Supplementary material for: Evaluation of Low-Cost Multi-Spectral Sensors for Measuring Chlorophyll Levels Across Diverse Leaf Types
Source: Sensors (Basel). 2025 Mar 31;25(7):2198. doi: 10.3390/s25072198 (PMC11991415; doi:10.3390/s25072198)
Supplement: Supplementary file 1 [file sensors-25-02198-s001.zip › Supplemental_Information_S1.pdf]

Device Characterization

The AS7265x sensors were assembled by SparkFun on breakout boards operating at a standard I^2C logic level of 3.3V, with power supplied by a SparkFun Qwiic cable. However, this voltage may not be sufficient to power the LEDs adequately. The Luxeon 3014 white LED on the board requires an operating voltage of 2.6 to 3.4V, while the Vishay UV LED operates between 2.8 to 3.8V. According to the AS7265x datasheet, the LED drive pin responsible for sinking the LED current must maintain a voltage above 0.3V. To assess compliance with this specification, the LED drive pin voltages were measured across the four current settings available on the AS7265x: 12.5, 25, 50, and 100 mA. This was done using an Analog Discover 2 USB oscilloscope (Digilent Inc., Pullman, WA, USA), along with its WaveForms software to measure the voltage of the current sink pins of the AS7265x chipset.

Figure S1 shows the measured voltages of the drive pins for the three LEDs: white (black line), IR (solid red line), and UV (blue line). The red dashed line in the figure indicates the 0.3V threshold specified in the AS7265x datasheet as the minimum required for the current sink. The results show that the white LED exceeds this threshold at 50 mA and 100 mA, while the UV LED consistently falls below the threshold, suggesting potential inaccuracies in current regulation at these settings. These findings suggest that the 3.3V power supply used by the AMS color sensors may be insufficient to power the white LED at high currents or the UV LED, which could be used for fluorescence experiments. Modifications to the board could include a separate voltage supply for the LEDs; however, care must be taken to ensure the LED drive pins do not exceed their maximum voltage rating of 3.3V.

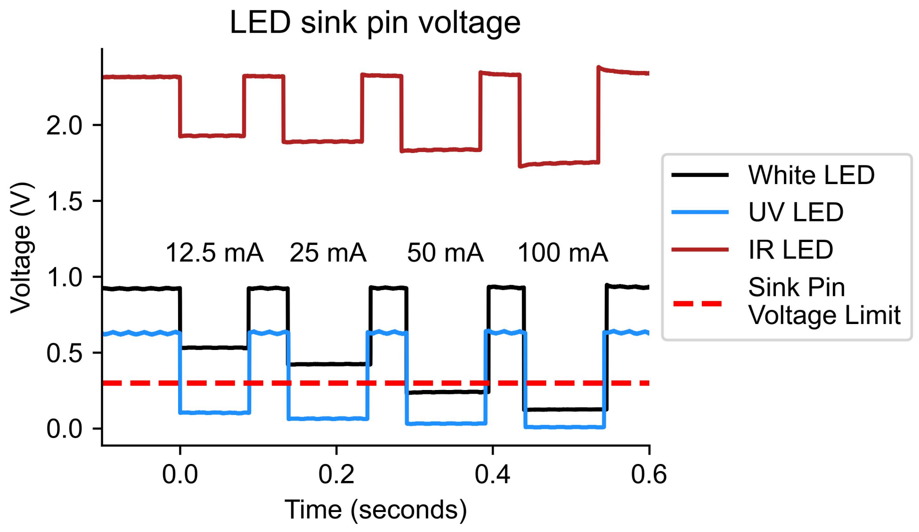

Figure S1: **LED drive pin voltage.** The measured voltage of the LED drive pins for the AS7265x chipset at four current settings: 12.5, 25, 50, and 100 mA. The white LED is shown in black, the IR LED in solid red, and the UV LED in blue. The red dashed line indicates the 0.3V threshold specified in the AS7265x datasheet as the minimum for the current sink.
