# Supplementary material for: Evaluation of Low-Cost Multi-Spectral Sensors for Measuring Chlorophyll Levels Across Diverse Leaf Types
Source: Sensors (Basel). 2025 Mar 31;25(7):2198. doi: 10.3390/s25072198 (PMC11991415; doi:10.3390/s25072198)
Supplement: Supplementary file 1 [file sensors-25-02198-s001.zip › Supplemental_Information_S2.pdf]

Extracted Chlorophyll Reference Measurements

Three hole punches from each leaf were taken to get the reference chlorophyll levels. Chlorophyll levels were determined using a destructive extraction method using dimethylformamide (DMF). To assess the precision of the chlorophyll measurements and identify outliers, the average chlorophyll level for each leaf was plotted against the three individual measurements (Figure S2a-e). Histograms of the residues (the differences between each individual measurement and the average for each leaf) are plotted in Figure S2f-j. A Gaussian distribution was fit to the residuals, and a 3-sigma threshold was applied to identify outliers. The individual measurement with the largest residual was removed, and a new average and residual for the leaf were calculated.

Figure S2a-e shows the removed outliers with a red cross, while the two remaining co-samples of the outlier are marked in green. When an outlier is removed, the co-samples move horizontally on the graph, indicated by the black arrows, as their average changes, but their individual measurements remain the same. 11 outliers were removed from 1,500 chlorophyll measurements. The R^2 scores of the individual measurements compared to the averages for the initial and final datasets are shown in the insets of Figure S2a-e, while the Mean Absolute Error (MAE) is displayed in the insets of Figure S2f-j. Both axes are uniformly scaled to allow comparison of the residual distributions. The chlorophyll data is available in the `data/chlorophyll` folder of the GitHub repository.

Outlier removal significantly improved R^2 scores for some leaves, notably jasmine, which increased from 0.79 to 0.93, and rice, which rose from 0.88 to 0.99. The final R^2 scores show that most leaves achieved an $R^2 \geq 0.98$, except for jasmine, which had an R^2 of 0.93 due to its non-uniform thickness and large venerations, making chlorophyll extraction more variable. Outlier removal reduced error levels across all datasets, with rice leaves showing a notable decrease in MAE from 1.81 to 0.81 μg/cm^2. The results reveal that mango, rice, and sugarcane leaves have narrow error distributions with MAE ranging from 0.81 to 1.00 μg/cm^2. In contrast, banana and jasmine leaves exhibit wider distributions with MAE values of 2.09 and 1.96 μg/cm^2, respectively.

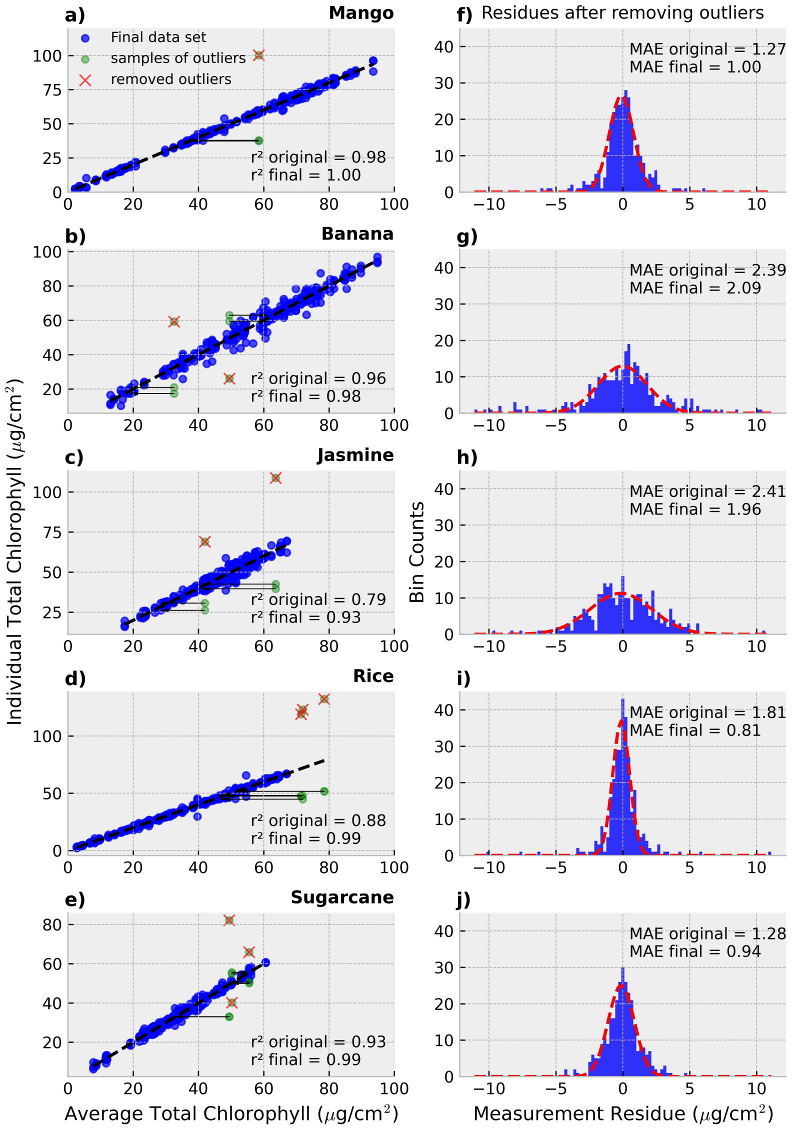

Figure S2: **Chlorophyll measurements.** (**a-e**) Plots of individual versus average leaf chlorophyll levels for the five leaves, as labeled in the figure. Removed outliers are shown with red crosses, and their associated co-samples are shown in green. Arrows indicate how the co-samples move when new average leaf chlorophyll levels are calculated. The remaining data points are shown in blue. The R^2 scores for the initial and final data sets are shown for each leaf. (**f-j**) Histogram of the residuals after removing outliers. Mean Absolute Errors (MAE) are shown for the initial and final data sets. All axes are set to the same scale to compare the precision of different leaves.
