# Supplementary material for: Evaluation of Low-Cost Multi-Spectral Sensors for Measuring Chlorophyll Levels Across Diverse Leaf Types
Source: Sensors (Basel). 2025 Mar 31;25(7):2198. doi: 10.3390/s25072198 (PMC11991415; doi:10.3390/s25072198)
Supplement: Supplementary file 1 [file sensors-25-02198-s001.zip › Supplemental_Information_S3.pdf]

Investigating Best Measurement Conditions

N-way analysis of variance (ANOVA) and pairwise post-hoc comparisons were performed using the Pingouin library in Python. Bonferroni correction was applied to correct p-values for multiple comparisons.

To evaluate these settings, N-way ANOVA was performed on the combined conditions, followed by pairwise comparisons for any significant factors. Four LED current settings, 12.5, 25, 50, and 100 mA, were tested, except for the AS7265x sensor, where only the first three were tested. Integration times of 140, 280, 420, 560, and 700 ms were tested for the AS7262 and AS7263 sensors, while only the three shorted times were tested for the AS7265x. Additionally, three data preparation methods, raw data, normalized reflectance, and absorbance, were applied to the models. The N-Way ANOVA also included leaf type to account for the variability between different leaves. N-way ANOVA results are shown in Tables S1–S3, with pairwise statistics provided in Appendix and summarized as violin plots in Figure S3. Uncorrected and Bonferroni-corrected p-values are included. Across all ANOVA tests, leaf type was the most significant in correlating chlorophyll levels.

Table S1 and Figure S3a, d, g display the ANOVA results for the AS7262 sensor, showing that the 12.5 mA LED current setting was statistically better than the other current settings. The poorer performance at 50 and 100 mA can be attributed to the saturation of the LED drive pin, as previously described. The better performance of 12.5 mA compared to 25 mA may be due to reduced thermal drift from lower power dissipation at lower currents, a phenomenon noted in a other reports. Neither integration time nor data type significantly affected the fitting results, although shorter integration times and converting the data to absorbance performed slightly better than other conditions.

Table S1: AS7262 ANOVA results for non-interacting components. Abbreviations: SS = Sum of Squares, DF = Degrees of Freedom, MS = Mean Square, F = F-ratio, p-unc = Uncorrected p-value, np2 = Partial eta-squared, p-corrected = Corrected p-value.

Source	SS	DF	MS	F	p-unc	np2	p-corrected
Leaf	9.46	4	2.36	585.63	$< \varepsilon$	4.65e-01	$< \varepsilon$
Measurement Type	0.01	2	0.01	1.76	0.172	1.30e-03	1.000
Integration Time	0.01	4	0.00	0.75	0.561	1.10e-03	1.000
LED Current	2.76	3	0.92	227.61	$< \varepsilon$	2.02e-01	$< \varepsilon$

Values smaller than the numerical tolerance ($\varepsilon \approx 2.22e-16$) are reported as $< \varepsilon$.

Table S2 and Figure S3b, e, h summarizes the statistical significance of the three parameters for the AS7263 sensor. Converting the data to absorbance was the most significant of the parameters. The 12.5 and 25 mA LED currents performed statistically similar but were better than 50 and 100 mA. Longer integration times showed statistically better performance than the two shortest

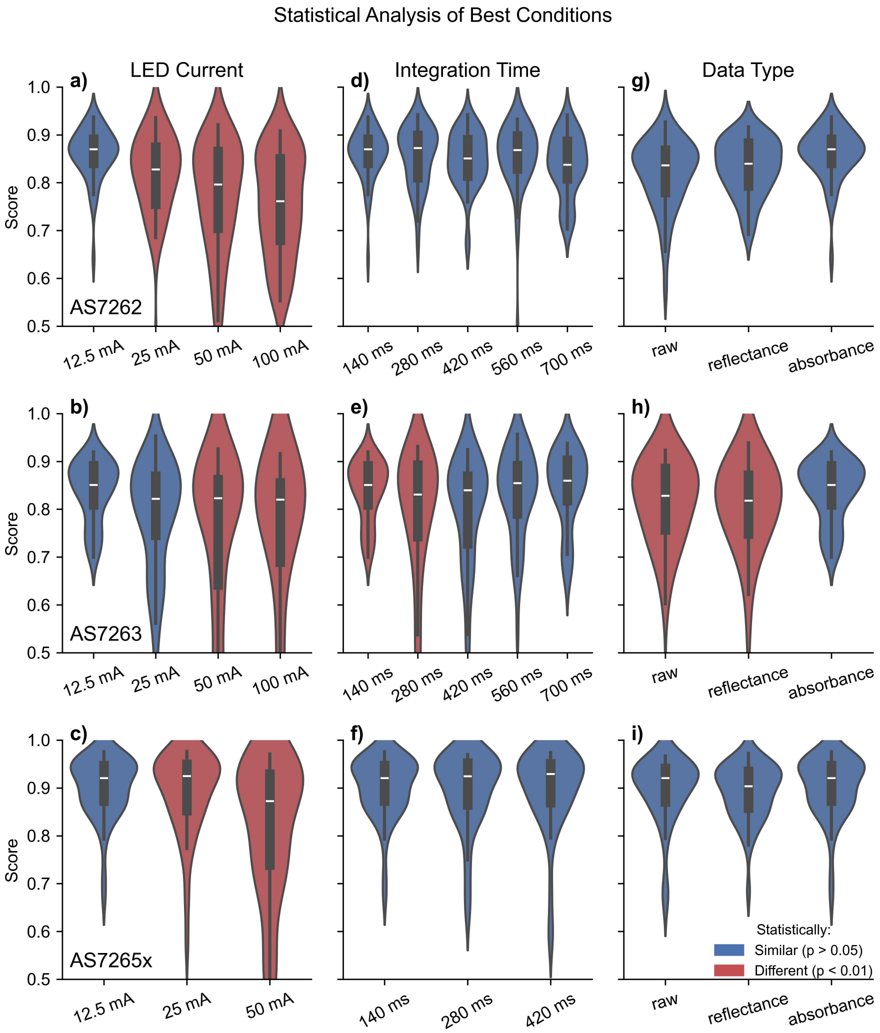

Figure S3: **Choosing best sensor conditions.** Results of ANOVA and pairwise comparison tests showing the best conditions and statistically similar results in blue (p ¿ 0.05) and statistically worse conditions in red (p¡0.01). Panels represent: (**a-c**) LED currents, (**d-f**) integration times, and (**g-i**) data preparation method for the sensors: (**a, d, g**) AS7262, (**b, e, h**) AS7263, and (**e, f, i**) AS7265x.

Table S2: AS7263 ANOVA results for non-interacting components. Definitions are as in Table 3.

Source	SS	DF	MS	F	p-unc	np2	p-corrected
Leaf	26.69	4	6.67	506.88	$< \varepsilon$	4.29e-01	$< \varepsilon$
Measurement Type	1.69	2	0.84	64.01	$< \varepsilon$	4.53e-02	$< \varepsilon$
Integration Time	1.04	4	0.26	19.76	4.79e-16	2.84e-02	7.18e-15
LED Current	0.95	3	0.32	24.14	2.06e-15	2.61e-02	3.09e-14

Values smaller than the numerical tolerance ($\varepsilon \approx 2.22e-16$) are reported as $< \varepsilon$.

times, contrasting with the AS7262 results. The integration times of 420, 560, and 700 ms were statistically similar, with 700 ms showing slightly better performance. These results likely reflect the sensor measuring in the NIR region using a visible LED, resulting in a smaller signal. Consequently, increasing integration time increases the signal size more than the noise introduced by thermal drift.

Table S3 and Figure S3c, f, i present the ANOVA results of the AS7265x sensor. The LED current statistically affected performance, with pairwise tests indicating that 12.5 mA was statistically better. ANOVA also indicated a significant effect of the integration time (p=0.017), though pairwise comparisons did not reveal specific differences. Additionally, ANOVA showed no statistical difference among absorbance, reflectance, or raw intensity for data fitting. This suggests the possibility of using a chlorophyll model using raw counts from the sensor, simplifying device programming and eliminating the need for reflectance measurements.

Table S3: AS7265x ANOVA results for non-interacting components. Definitions are as in Table 3.

Source	SS	DF	MS	F	p-unc	np2	p-corrected
Leaf	6.28	4	1.57	388.08	$< \varepsilon$	5.61e-01	$< \varepsilon$
Measurement Type	0.01	2	0.00	0.78	0.459	1.28e-03	1.000
Integration Time	0.06	2	0.03	6.85	1.10e-03	1.12e-02	0.017
LED Current	1.62	2	0.81	200.04	$< \varepsilon$	2.48e-01	$< \varepsilon$

Values smaller than the numerical tolerance ($\varepsilon \approx 2.22e-16$) are reported as $< \varepsilon$.

The ANOVA and pairwise comparison tests showed that the lowest current of 12.5 mA performed the best for all devices and is optimal for design purposes as it minimizes current and power consumption. Converting the data to absorbance yielded the best results for all sensors, although this was not statistically significant for the AS7262 and AS7265x. The shortest integration time was chosen for the AS7262 and AS7265x as it performed the best, while 700 ms was selected for the AS7263 for optimal performance. This parameter selection process was iterative, starting with the shortest integration time and lowest

current to perform initial PLS scans for determining the number of LVs before performing statistical tests. After statistically checking the best conditions, the process was repeated for the AS7263 with a 700 ms integration time. All figures in the main text use a 700 ms integration time for the AS7263 and 140 ms for the other sensors.

Pairwise Comparisons

Table S4: AS7262 LED Current Pairwise tests. Abbreviations: Contrast = Comparison of groups, A = Group A, B = Group B, T = Test statistic, p-unc = Uncorrected p-value, p-corr = Corrected p-value, BF10 = Bayes Factor for evidence in favor of the alternative hypothesis.

Contrast	A	B	T	p-unc	p-corr	BF10
LED Current	100 mA	12.5 mA	-17.27	$< \varepsilon$	$< \varepsilon$	2.9e+57
LED Current	100 mA	25 mA	-11.05	$< \varepsilon$	$< \varepsilon$	1.159e+24
LED Current	100 mA	50 mA	-2.84	4.63e-03	0.028	3.077
LED Current	12.5 mA	25 mA	5.43	6.40e-08	3.84e-07	1.139e+05
LED Current	12.5 mA	50 mA	14.62	$< \varepsilon$	$< \varepsilon$	6.994e+41
LED Current	25 mA	50 mA	8.40	$< \varepsilon$	6.05e-16	4.12e+13

Values smaller than the numerical tolerance ($\varepsilon \approx 2.22 \times 10^{-16}$) are reported as $< \varepsilon$.

Table S5: AS7263 Measurement Type Pairwise tests. Definitions are as in Table S1.

Contrast	A	B	T	p-unc	p-corr	BF10
Measurement Type	absorbance	raw	7.54	6.92e-14	2.07e-13	6.353e+10
Measurement Type	absorbance	reflectance	7.50	9.75e-14	2.93e-13	4.54e+10
Measurement Type	raw	reflectance	-0.01	0.995	1.000	0.05

Table S8: AS7265x Integration Time Pairwise tests. Definitions are as in Table S1.

Contrast	A	B	T	p-unc	p-corr	BF10
Integration Time	50	100	1.77	0.077	0.231	0.348
Integration Time	50	150	-0.23	0.815	1.000	0.077
Integration Time	100	150	-2.04	0.041	0.124	0.578

Table S6: AS7263 Integration Time Pairwise tests. Definitions are as in Table S1.

Contrast	A	B	T	p-unc	p-corr	BF10
Integration Time	50	100	-1.48	0.140	1.000	0.19
Integration Time	50	150	-2.49	0.013	0.129	1.372
Integration Time	50	200	-5.18	2.54e-07	2.54e-06	3.297e+04
Integration Time	50	250	-5.01	6.35e-07	6.35e-06	1.375e+04
Integration Time	100	150	-1.18	0.238	1.000	0.129
Integration Time	100	200	-3.95	8.13e-05	8.13e-04	139.779
Integration Time	100	250	-3.82	1.40e-04	1.40e-03	83.917
Integration Time	150	200	-2.45	0.015	0.146	1.232
Integration Time	150	250	-2.41	0.016	0.159	1.143
Integration Time	200	250	-0.10	0.920	1.000	0.065

Table S7: AS7263 LED Current Pairwise tests. Definitions are as in Table S1.

Contrast	A	B	T	p-unc	p-corr	BF10
LED Current	100 mA	12.5 mA	-4.76	2.13e-06	1.28e-05	3974.705
LED Current	100 mA	25 mA	-4.20	2.77e-05	1.66e-04	349.315
LED Current	100 mA	50 mA	-0.49	0.625	1.000	0.065
LED Current	12.5 mA	25 mA	0.59	0.553	1.000	0.069
LED Current	12.5 mA	50 mA	4.42	1.05e-05	6.27e-05	878.189
LED Current	25 mA	50 mA	3.84	1.26e-04	7.57e-04	84.268

Table S9: AS7265x LED Current Pairwise tests. Definitions are as in Table S1.

Contrast	A	B	T	p-unc	p-corr	BF10
LED Current	12.5 mA	25 mA	3.89	1.08e-04	3.24e-04	120.518
LED Current	12.5 mA	50 mA	11.97	$< \varepsilon$	$< \varepsilon$	2.618e+27
LED Current	25 mA	50 mA	8.31	3.45e-16	1.04e-15	1.379e+13

Values smaller than the numerical tolerance ($\varepsilon \approx 2.22 \times 10^{-16}$) are reported as $< \varepsilon$.
