# Supplementary material for: Evaluation of Low-Cost Multi-Spectral Sensors for Measuring Chlorophyll Levels Across Diverse Leaf Types
Source: Sensors (Basel). 2025 Mar 31;25(7):2198. doi: 10.3390/s25072198 (PMC11991415; doi:10.3390/s25072198)
Supplement: Supplementary file 1 [file sensors-25-02198-s001.zip › Supplemental_Information_S4.pdf]

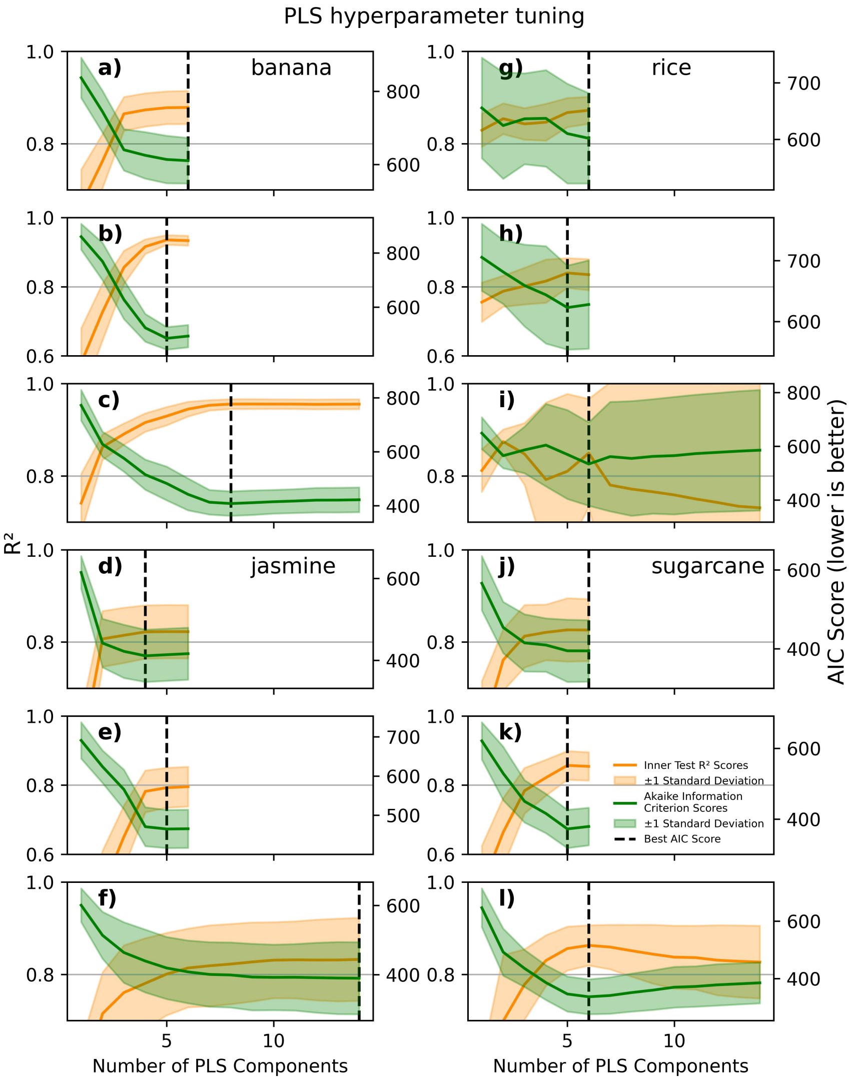

Figure S4: **Optimal number of Latent Variables.** Choosing the optimal number of LVs for the PLS models of remaining leaves for the sensors: (**a, d, g, j**) AS7262, (**b, e, h, k**) AS7263, and (**c, f, I, l**) AS7265x, for the leaves: (**a-c**) banana, (**d-f**) jasmine, (**g-i**) rice, and (**j-l**) sugarcane. The best number of LVs is based on the minimum AIC score (green line). Inner CV R^2 test scores are in orange, with standard deviations as shaded regions, and the minimum AIC score is indicated with the vertical dashed black line.
